# Gun Violence and Firearm Injuries in West Michigan: Targeting Prevention

**DOI:** 10.5811/westjem.2021.3.49255

**Published:** 2021-05-19

**Authors:** Christopher M. Mattson, Ryan Kaylor, Tracy J. Koehler, Marc Ydenberg, Justin Grill, Brian R. Stork

**Affiliations:** * Mercy Health, Department of Emergency Medicine, Muskegon, Michigan; † Naval Medical Center, Department of Emergency Medicine, San Diego, California; ‡ Mercy Health, Department of Scholarly Activity Support, Muskegon, Michigan; § University of Michigan, Department of Urology, Ann Arbor, Michigan

## Abstract

**Introduction:**

Firearm-related deaths and injuries are ongoing public health issues in the United States. We reviewed a series of gun violence- and firearm-related injuries treated at a multi-campus community healthcare system in West Michigan to better understand the demographic and clinical characteristics of these injuries. We also studied hospital charges, and payers responsible, in an effort to identify stakeholders and opportunities for community- and hospital-based prevention.

**Methods:**

We performed a retrospective review of firearm injuries treated at Mercy Health Muskegon (MHM) between May 1, 2015 and June 30, 2019. Demographic data, injury type, Injury Severity Score (ISS), anatomic location and organ systems involved, length of stay (LOS), mortality, time of year, and ZIP code in which the injury occurred were reviewed, as were hospital charges and payers responsible.

**Results:**

Of those reviewed, 307 firearm-related injuries met inclusion criteria for the study. In 69.4% of cases the injury type was attempted murder or intent to do bodily harm. Accidental and self-inflicted injuries accounted for 25% of cases. There was a statistically significant difference in the mechanism of injury between Black and White patients with a higher proportion of Black men injured due to gun violence (P < 0.001). Median ISS was 8 and the most commonly injured organ system was musculoskeletal. Median LOS was one day. Self-inflicted firearm injuries had the highest rate of mortality (50%) followed by attempted murder (7%) and accidental discharge (3.1%; P < 0.001). Median hospital charge was $8,008. In 68% of cases, Medicaid was the payer. MHM received $4.98 million dollars in reimbursement from Medicaid; however, when direct and indirect costs were taken into account, a loss of $12,648 was observed.

**Conclusion:**

Findings from this study reveal that young, Black men are the primary victims of gun violence-related injuries in our West Michigan service area. Hospital care of firearm-related injuries at MHM was predominantly paid for by Medicaid. Multiple stakeholders stand to benefit from funding and supporting community- and hospital-based prevention programs designed to reduce gun violence and firearm-related injuries in our service area.

## INTRODUCTION

Firearm-related deaths and injuries are ongoing public health issues in the United States (US) and in West Michigan. The increasing frequency of these events, most recently in prominent cities such as Atlanta, GA and Boulder, CO, has placed a growing toll on communities nationwide, both in terms of morbidity and mortality, and monetary cost.^1^ While mass casualty incidents have historically received the majority of media attention, fatalities in mass shooting incidents in the US account for only a fraction of all gun murders that occur nationwide each year.^2^–^4^ If we define mass casualty incidents as those events involving four or more victims (excluding the shooter), there were 373 reported deaths in 2018.^2^ Overall, between 2010–2016 there were more than 595,000 injuries reportedly caused by firearms in the US.^5^,^6^ During that same period, firearms were involved in 8133 deaths in the state of Michigan.^7^

Mercy Health Muskegon (MHM) is a community-based healthcare system located in West Michigan. A member of Trinity Health, MHM through its three hospital campuses provides an estimated 90% of healthcare services to the region it serves.^8^ Each campus, by way of its associated emergency department (ED), serves a unique patient population. The system’s Hackley and Sherman campuses, for example, serve inner city, suburban, and rural populations. They also accept transfer patients from other hospitals. Alternatively, the Lakeshore campus predominantly serves a rural population. Mercy Health Muskegon established a Level II trauma center on its Hackley Campus on May 1, 2015. The opening of this center has resulted in numerous benefits to the surrounding communities, including having 24-hour access to multiple specialties, a dedicated trauma service, and a trauma coordinator to assist with quality improvement and outcome reporting.

Our goal in this study was to describe the demographic, clinical, and situational characteristics of firearm injuries, as well as outcomes, hospital charges, and payers. This information could be used to support future resource allocation and firearm-related injury prevention efforts.

## METHODS

**Figure 1:**
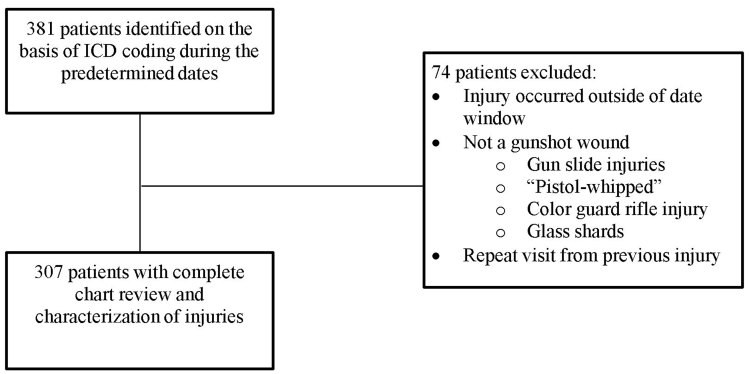
Inclusion flow chart.

After obtaining approval from the Mercy Health Grand Rapids Institutional Review Board, we performed a retrospective review of all firearm-related injuries treated at MHM hospital EDs between May 1, 2015–June 30, 2019. These hospitals included Mercy Health Lakeshore Campus, Mercy Health Muskegon Campus, and Mercy Health Hackley Campus. A start date of May 1, 2015, was chosen because it was the first day Mercy Health Hackley Campus began servicing the community as a Level II trauma center. For the purpose of this study, we defined firearm injuries as any injury resulting from the discharge of a firearm with penetration or abrasion to the subject’s body by the projectile. We used preselected *International Classification of Diseases*, revisions 9 and 10 (ICD)-9 and ICD-10 diagnostic codes (Supplement 1) to query the hospital charges database to identify patients. After identifying potential charts, two investigators (CM and RK) independently reviewed each patient’s chart to ensure it met criteria for inclusion (Figure 1). Of the 381 cases identified by ICD coding, 74 cases were excluded.

Study variables included the following: age; gender; race; mechanism of injury (e.g., attempted murder, accidental, self-inflicted); firearm involved; method of arrival to hospital (e.g., ambulance, car); Injury Severity Score (ISS); anatomic location(s) of injury(ies); organ system(s) affected; comorbidities requiring treatment during that visit/stay; length of stay (LOS); mortality; time of year (month); geographic region of injury (ZIP code); hospital charges; and payer. For the purposes of this study, hospital charges included only fees charged by the hospital itself. Other charges related to patient care, such as professional fees charged by emergency physicians, anesthesiologists, and radiologists in private practice, were not captured. Also omitted were charges associated with after-visit care at other facilities (e.g., acute rehabilitation stays, physical therapy visits).

**Population Health Research Capsule**

What do we already know about this issue?

*Firearm related deaths and injuries are a major public health issue in the United States. Though more heavily publicized, mass shootings make up a minority of these events.*

What was the research question?

*Our goal in this study was to describe the demographic, clinical, and situational characteristics of firearm injuries in our community, as well as analyze outcomes, review hospital charges, and track payers.*

What was the major finding of the study?

*Hospital-based care of gun violence injuries in our community is resource intensive, leads to significant Medicare expenditures, and results in a net loss of revenue for our health care system.*

How does this improve population health?

*These findings will support future resource allocation and firearm-related injury prevention efforts in the community.*

We calculated summary statistics for the data. Quantitative data are shown as mean ± standard deviation or median and interquartile range (IQR) or minimum/maximum values for non-normally distributed variables. Nominal data are shown as percentages. Quantitative data were compared using the Kruskal-Wallis test and nominal variables were compared using the chi-square or Fisher’s exact test when appropriate. We analyzed data using SPSS Statistics, v. 23 (IBM Corp., Armonk, NY).

## RESULTS

### Patient and Clinical Characteristics

A total of 307 firearm-related injuries met inclusion criteria for the study. Table 1 shows the demographic, clinical, and firearm injury characteristics of our subjects. The average age was 27.2±12.9 years, and patients were predominantly male. Blacks accounted for more than 70% of injuries. Median ISS was 8 ([IQR: 1–15], n = 165), and less than 10% of patients had other medical comorbidities treated concurrently. The median ISS was significantly lower for injuries related to accidental discharge (1 [IQR: 1–9.3]) when compared to self-inflicted wounds (21 [IQR: 6.5–25]; *P* = 0.002) as well as between accidental discharge and attempted murder/bodily harm (9 [IQR: 2.5–14]; *P* = 0.03. Table 2 depicts comparisons by mechanism of injury.

### Injury-Related Characteristics

**Figure 2:**
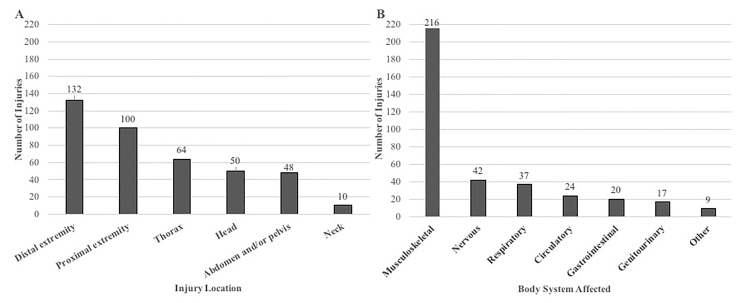
A) Firearm injury frequency by body area(s); B) body system(s) affected.

Nearly 70% of injuries were the result of attempted murder and were due to single rather than multiple gunshot wounds. There was a statistically significant difference in the rates of mechanism of injury between Black and White patients (*P* <0.001). A higher proportion of Blacks were injured due to attempted murder, compared with Whites (85% vs 40%), whereas rates of accidental discharge and self-inflicted injuries were higher in White patients (47% vs 12.7% and 13% vs 2%, respectively). Patients with self-inflicted injuries were significantly older than patients with injuries from an attempted murder or bodily harm (39 [25.7–62] vs 25 [19–31.5]; *P* = 0.011), as well as for self-inflicted injuries and accidental discharge (22 [17.3–34.8]; *P* = 0.008; Table 2). Handguns were the most common type of weapon used; however, weapon type was documented in only 33% of cases. Mode of transportation to the ED was split closely between private vehicle/walk-in and ambulance arrival. Injury location and body system involved are shown in Figure 2A and 2B. The majority of injuries were to the distal extremities. Musculoskeletal injuries accounted for the bulk of cases (70%), ranging from compound fractures to mild musculoskeletal tears. Other organ system injuries occurred much less frequently.

### Mortality, Length of Stay, and Hospital Charges

Tables 2 and 3 show results related to mechanism of injury comparisons and overall LOS, survival, hospital charges, and payers, respectively. More than 90% of visits related to firearm injuries were non-fatal, resulting in a median LOS of one day. Self-inflicted firearm injuries had the highest rate of mortality (50%) compared with attempted murder (7%) and accidental discharge (3.1%; *P* < 0.001). Median LOS in survivors was significantly different between injuries related to accidental discharge compared with self-inflicted (1 [IQR: 1–1] vs 2 [IQR: 1–3]; *P* = 0.007, respectively) as well as between accidental discharge injuries and attempted murder/bodily harm (1 [IQR: 1–3]; *P* < 0.001). Of the 26 fatalities, 19 were due to a non-self-inflicted cause, and 7 were attributed to the victims themselves. Total hospital charges for patients treated for firearm-related injuries were $6.37 million.Median hospital charge was $8,008 [IQR: $2,024 –$21,716]. Median charges were significantly lower for accidental injuries compared with attempted murder/bodily harm injuries ($1381 [IQR: $825–$10,041] vs $10,184 [IQR: 3314–$31,250]; *P* < 0.001) and self-inflicted injuries ($19,508 [IQR:10,849–$25,921]; *P* < 0.001). Hospital reimbursement for the care of the majority of patients (67.8%) was provided by Medicaid. When direct and indirect costs were taken into consideration, MHM reported a $12,648 loss on the care of these patients (Nagengast, CPA, FHFMA, and C. Kosheba [personal communication, July 27, 2020]).

### Time of Year and Region

**Figure 3:**
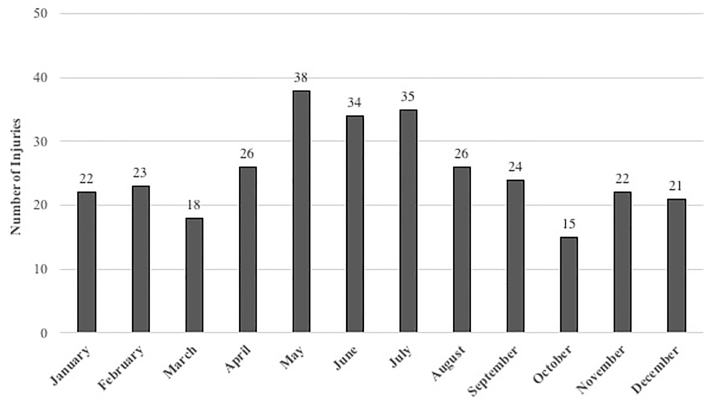
Frequency of firearm injuries in Michigan by month for study period.

The number of firearm injuries by time of year is shown in Figure 3. Most occurred during the summer months. When comparing by time of year (e.g., winter: December–February; spring: March–May; summer: June–August; and fall: September–November) this trend was not statistically significant (*P* = 0.54; Table 2). Of injuries recorded, 79% occurred within two ZIP codes, which included the cities of Muskegon and Muskegon Heights.

## DISCUSSION

Our results showed more than 90% of visits related to firearm injuries were non-fatal, with ISS scores on the lower end resulting in a median LOS of one day. This appears to be the result of numerous superficial or distal injuries not requiring prolonged (or any) hospitalization. Many patients were discharged home on the same day as their presentation to the ED. Most injuries occurred within two ZIP codes served by our hospital system with the majority occurring during the warmer months of the year. Characteristics of the patient population and mechanism of injury included high rates of attempted murder/bodily injury involving Black males. These findings are similar to previous demographic studies of gun violence injuries in other communities.^1^,^9^,^10^ Accidental discharge injuries were associated with lower ISS, LOS, and hospital charges, whereas self-inflicted injuries occurred mainly in older adults and were more expensive with higher mortality rates.

### Violent Crime

The high incidence of firearm-related injuries has received intense scrutiny throughout the nation. In 2018, firearm-related violence made up 26.1% of all aggravated assaults in the United States.^11^ Recently, gun violence has again erupted in cities such as Atlanta, GA and Boulder, CO, highlighting the continued relevance. During our defined study period, Michigan State Police reported 618 cases involving a firearm in Muskegon County, 36 of which resulted in death.^12^ As a result, Mercy Health EDs are frequently charged with caring for the victims of firearm injuries.

Blacks were victims of 73% of all firearm-related injuries during the study period. Furthermore, of the 226 events where ZIP code was recorded, we found 80% were clustered within 49442 and 49444. These ZIP codes include the cities of Muskegon and Muskegon Heights. A 2016 FBI statistics report showed the 49442 and 49444 ZIP codes were home to some of the highest violent crimes per capita in the state.^13^ These same areas have a 74.5% Black population with a poverty rate of 37.9% (national poverty rate estimated to be approximately 15.7%).^14^ The Muskegon County population (containing both cities previously described) is estimated to be 81.2% White, 14% Black, and 5.8% Hispanic or Latino, for comparison.^14^ Multiple peer-reviewed sources note that individuals suffering from low socioeconomic status are at increased risk for both committing and being victims of violent crime.^15^,^16^ The apparent racial disparity appears to be related to socioeconomic conditions and increased poverty rates in the local Black community, particularly in these areas.

Researchers have attempted to identify individuals who are at increased risk for interpersonal violence. Goldstick et al developed the SaFETY score as a way to predict future firearm violence. This risk-stratification tool identifies very high-risk individuals (e.g., those with a SaFETY score > 5) who are likely candidates for entry into resource-intensive programs.^17^ Similarly, Kramer et al^18^ established an algorithmic tool to predict violent reinjury, the “Violent Reinjury Risk Assessment Instrument,” which could help with resource allocation.

In addition to risk stratification, the Flint Youth Injury Study noted a strong relationship between substance use and violence among a high-risk urban minority sample.^19^ Addressing substance use and poverty and improving the socioeconomic status of all American ethnic groups should be of paramount importance. This may require a significant amount of government and private aid in combination with public policy reform over several years and perhaps even decades. A better short-term solution may be to address gaps in public education and to provide more outreach programs.^19^,^20^

The American Association for the Surgery of Trauma Prevention Committee recommends hospital-based violence intervention programs (HVIP) as a means of reducing interpersonal violence.^21^ Throughout the country, physicians and hospital systems have joined the effort to help reduce gun violence in their respective communities with some success.^1^,^22^ Between 1999–2001, for example, the R. Adams Cowley Shock Trauma Center in Baltimore, MD, implemented and reviewed a HVIP.^23^ This model used a multidisciplinary approach, including conflict resolution and public safety issues, recovery from injury, development of positive skills/support, and connection to community services. The center was able to demonstrate a firearm injury recidivism rate for program participants of 5%, compared with a 36% recidivism rate for the control group not receiving violence intervention services, which translated to a cost difference of $598,000 between groups.^23^

Two additional HVIPs, Within Our Reach and the Wraparound Project, tested a varying degree of social services to prevent re-injury in patients. The first program used a control group that was provided simply a written list of services, whereas the treatment group received an assessment and case management for six months. Both groups were evaluated at six and 12 months after enrollment in the study; overall they noted a 12.2% reduction in self-reported re-injury in the intervention group (20.4% vs 8.1%).^24^ The latter project focused on meeting the needs of patients in two specific domains: mental health and employment. In their HVIP, they were able to demonstrate a recidivism rate of 4.5% vs the historical control of 16%.^25^

Prescription for Hope (RxH) took a unique approach: RxH support specialists conduct an in-depth assessment of patients admitted with a violent injury. They provide a tailored plan with a multitude of community services and after analysis of eight years of data demonstrated a 4.4% recidivism rate among program participants.^26^

The cities of Muskegon and Muskegon Heights have also taken steps to address the gun violence in parts of their cities. For example, in June 2019 a local fundraiser supported by police departments in Muskegon County and Meijer, Inc., created the first annual Guns for Groceries Community Health and Safety Day. This “no questions asked” program allowed citizens to exchange any type of weapon, to be appropriately disposed of by the Muskegon Heights Police Department, for a $100 grocery gift card. It was reported that 137 guns, ranging from rifles to shotguns were collected.^27^ That same month, religious, community, and business leaders began a series of town hall meetings called Gaining Unity Through Non-Violent Solutions or G.U.N.S. These meetings served as an opportunity for community members to think about and openly discuss ways that they could work together to better support at-risk youth and reduce violence.^28^ In 2019, G.U.N.S. held a fundraising basketball game in conjunction with local law enforcement to help increase awareness in the community. The event was so successful that the organization planned to make it an annual event.^29^

Our study data, combined with grass root efforts ongoing in the MHM service area and the fact that successful, healthcare-led prevention programs already exist in other cities, suggest that a physician-led, hospital-based program and clinical screening tool to reduce gun violence would further benefit our community. This would have the potential to not only improve the health and safety of at-risk persons in our service area, but also reduce preventable healthcare utilization and costs.

### Accidental Injury

Within the study period there were 65 firearm injuries classified as “accidental injuries.” The bulk of this group was made up of young (average age 28), White (57.1%) males (78.1%). These specific types of injuries carried a low mortality rate of only 3.1%. Reasons for gun ownership in Michigan vary from person to person, including protection/safety, hunting, sport shooting, collector pieces, and vocational requirements. Limited reporting prevented our ability to statistically evaluate the events and mechanisms that caused these “accidental injuries”; however, common accidents we found included self-inflicted injury from mishandling a weapon (cleaning, loading, or playing with the weapon) and hunting/sport shooting accidents.

Currently, several organizations offer firearm training courses, some free to the public, in and around Muskegon County. These gun safety courses teach general firearm safety rules: how to safely store your weapon; the fundamentals of holding, loading, and shooting the weapon; and some courses provide combat preparation for high-stress situations. Further gun safety and training outreach should be considered for the local communities of West Michigan to reduce the number of “accidental injuries” from firearms. Most, if not all, cases are preventable with better knowledge and safety precautions.^30^

In addition, due to the plentiful game and numerous opportunities for hunting in the state of Michigan, there are a large number of registered hunters. As mentioned above, hunting and hunting-related activities are potential causes for firearm-related deaths and injuries. In contrast to our expectations, only a handful of cases were attributed to hunting-related activities in our study group. In general, the MHM ED sees few hunting-related accidents. This could be attributed to the fact that hunting in this area is often a family activity, where there is supervision from a parent or guardian. Credit could also be given to state regulations mandating that all new hunters born on or after January 1, 1960, must obtain a “hunter safety certificate.”^31^ According to Michigan’s Department of Natural Resources (DNR, in the 10 years leading up to 2019, there were only 20 hunting-related fatalities in Michigan and 122 injuries. The DNR has tracked a steady decline in firearm-injury incidents since 1977, when they began to require hunters to wear orange in the field and improved safety courses.^32^

Another confounding variable increasing “accidental firearm injuries” is the mishandling by unregistered users, specifically children who gain access to unsecured weapons. A 2005 study showed that locking up firearms and ammunition reduced the risk of self-inflicted firearm injury by 78%, and lowered risk of accidental pediatric firearm injury by 85% compared with no intervention.^30^ Another study in 2019 estimated that if half of households with children attempted to lock up their firearms, up to one third of youth gun suicide and accidental deaths could be prevented.^33^ Currently there is a national ad campaign called “End Family Fire,” endorsed by at least 25 different organizations, whose aim is to decrease the number of incidents of accidental firearm injury/death related to inadequate safe gun-storage practices.^34^ Another impressive resource is “Project Child Safe,” a program supported by the National Shooting Sports Foundation.^35^ They partner with local law enforcement throughout the nation to provide free cable-style gun locks with safety instructions to better secure one’s firarms.

### Intentional Self-inflicted Injury

There were 14 “intentional or self-inflicted” firearm-injury cases reported in our study group. The majority of these injuries occurred in White (71.4%) men (78.6%), with a mean age of 42.5 years old. Seven cases, or 50%, resulted in mortality for the victim. The mortality rate in this group was the highest when compared with all other firearm injury groups.

According to aggregated data from the Centers for Disease Control and Prevention, in 2017 the rate of suicide in the US was approximately 14 victims per 100,000 persons.^36^ This equated to roughly 42,700 suicides across the nation that year.^36^ Moreover, the rate of suicide within the State of Michigan was also 14.1 victims per 100,000 persons.^37^ Although attempted murder and homicide often make headlines, in most counties in Michigan it is actually suicide and suicide attempts that make up the largest number of firearm-related injuries. Between 2008–2013, for example, only three counties in Michigan reported more homicides than suicides.^38^ For our purposes, the county of Muskegon reported a much higher suicide rate than the national average at roughly 17.9 victims per 100,000 persons.^37^ This equated to 71 suicides by firearm in the county between 2015–2019.^39^ Initially we found it difficult to explain why, given the higher than average suicide rate in our community, we were seeing so few firearm-related suicide victims in our EDs. After speaking with local law enforcement officials we now believe this is likely due to the fact that suicide attempts involving guns are very often fatal and that these patients many times die outside of the hospital and never actually make it to the ED.^40^

The high rate of “self-inflicted injuries” and mortality associated with these injuries in Muskegon County is distressing; however, local data-driven groups such as the Muskegon County Suicide Prevention Coalition are actively working to reverse this trend. Beginning in 2006, these groups crafted a broad plan to reduce overall deaths by suicide. Their guiding principles are to promote awareness, reduce stigma and barriers, increase protective factors and reduce risk factors, promote community resources, and to be data driven.^41^ To improve suicide prevention and gun safety, they are working with community leaders and healthcare officials in Muskegon to implement outreach programs.^42^ For example, there is free online training for healthcare professionals called CALM (counseling on access to lethal means) provided through the Suicide Prevention Resource Center. This educational course helps providers identify red flags and reduce the access to lethal means, such as firearms and medications.^43^

## LIMITATIONS

We used ICD-9 and -10 codes, specific to firearm-related injuries, to collect cases that occurred at MHM and its Level II trauma center. Cases that were mislabeled or coded with an alternative ICD 9/10 code may not have been captured. Neither did we capture the number of individuals who suffered mortality before transport. In addition, the type of weapon involved was only documented 33% of the time. On the basis of electronic health record charting alone, it is difficult to make any definitive statements about the types of firearms responsible for injuries in our community. Further investigation and an emphasis on improving provider documentation of weapon type is recommended. Injury Severity Score data were recorded in only 53.7% of cases. This may be due in part to the fact that in accordance with the hospitals’ trauma registry inclusion criteria, injury scores were not calculated for patients who were treated and discharged directly from the ED (M. Kucera RN, BSN, Trauma Program Manager, [personal communication, January 5, 2021]).

In addition, we used hospital charges to the patient/insurer to quantify economic burden. It should be noted that analyzing hospital charges alone does not properly represent the total burden to each patient. This total omits bills issued by private providers and groups (anesthesiologists, radiologists, emergency physicians, etc) or private ambulance services. Furthermore, it does not include any costs incurred after discharge from the hospital, which include acute rehabilitation, visiting nurses, and physical therapy. Victims’ legal fees and lost income/wages as a result of injuries sustained from a firearm were not a part of this study.

We obtained the data in this study from three different MHM EDs in West Michigan. As the frequency and nature of gun violence can vary significantly by community, the results of this study may not be generalizable to other EDs, hospitals, or communities.

## CONCLUSION

In this study, younger Black males were identified to be the primary victims of gun violence-related injuries in our service area. Hospital visits for these injuries were associated with a net monetary loss for the hospital system and high burden to Medicaid. Review of the literature supports a multi-disciplinary approach to firearm-related injury reduction and costs associated with their care. Hospital-based intervention programs partnered with community resources are an effective tool for injury recidivism and cost reduction. Moving forward, the institution of a hospital-based intervention program with emphasis on the identified high-risk population offers an opportunity to help prevent recurrent injury and decrease financial costs for the system.

## Supplementary Information





## Figures and Tables

**Table 1:** Demographic, clinical and firearm injury characteristics, N = 307.

Characteristic	No. (%)^
Age, years*	27.7 ± 12.9
Gender	
Male	273 (88.9)
Female	34 (11.1)
Race	
Black	224 (73.0)
White	81 (26.4)
Multiracial	2 (0.7)
Ethnicity	
Hispanic/Latino	9 (2.9)
Injury Severity Score, (n = 165)#	8 [1–15]
Patients receiving treatment for comorbidities during management of firearm injury	17 (5.5)
Mechanism of injury	
Attempted murder	213 (69.4)
Accidental discharge/mishandling of a weapon	64 (20.8)
Other/unknown	16 (5.2)
Self-inflicted	14 (4.6)
Mechanism of arrival (n = 304)	
Ambulance	154 (50.7)
Private vehicle/walking	150 (48.9)
Previous gun injury	18 (5.9)
Projectile number	
Single gunshot	240 (78.2)
Multiple gunshots	67 (21.8)
Weapon type (n = 99)	
Handgun	61 (61.6)
Shotgun	6 (6.1)
Long gun	3 (3)
BB gun/air gun	29 (29.3)
Treating location	
Hackley Hospital (inner city)	253 (82.4)
Mercy Health Muskegon (inner city)	46 (15)
Lakeshore Hospital (rural)	8 (2.6)

^ Unless otherwise noted.

* Mean ± standard deviation.

# Median [interquartile range].

**Table 2:** Mechanism of injury comparisons.

Characteristic	Attempted murder/bodily harm n=213	Accidental discharge n=64	Self-inflicted n=14	*P*-value
Age*	25 [19–31.5]^#^	22 [17.3–34.8]^^^	39 [25.7–62]^#^^	0.011^#^; 0.008^^^
Race, No. (%)				
Black	182 (85.4)	27 (12.7)	4 (1.9)	<0.001
White	31 (40.3)	36 (46.8)	10 (13)	
Injury Severity Score*	9 [2.5–14]^#^ n=124	1 [1–9.3]^#^^ n=18	21 [6.5–25]^^^ n=12	0.03^#^; 0.002^^^
LOS, survivors	1 [1–3]^#^ n=198	1 [1-1]^#^^ n=62	2 [1–3]^^^ n=7	<0.001^#^; 0.007^^^
Mortality, No. (%)	15 (7)	2 (3.1)	7 (50)	<0.001
Hospital charges*	$10,184 [$3,314–$31,250]	$1381 [$825–$10,041]	$19,508 [$10,849–$25,921]	<0.001
Time of year, No. (%)				
Fall	43 (20.4)	11 (17.2)	2 (14.3)	0.54
Spring	61 (28.9)	17 (26.6)	2 (14.3)	
Summer	62 (29.4)	24 (37.5)	4 (28.6)	
Winter	45 (21.3)	12 (18.8)	6 (42.9)	

* Median [interquartile range].

Superscripts #,^ denote the comparison between columns and their associated significant *P*-value.

*LOS*, length of stay.

**Table 3:** Outcomes, payer and cost information, N = 307.

Outcome	Value
Length of stay, days#	1 (1–29)
Mortality, No. (%)	26 (8.5)

Payer	No. (%); total charges

Public aid	208 (67.8%); $4,979,964
Commercial insurance	30 (9.8%); $447,875
Blue Cross/Blue Shield	27 (8.8%); $344,555
Uninsured	24 (7.8%); $283,624
Medicare	15 (4.9%); $283,394
Other	3 (1.0%); $30,303

# Median (minimum – maximum values).
