# Supplementary material for: Gun Violence and Firearm Injuries in West Michigan: Targeting Prevention
Source: West J Emerg Med. 2021 May 19;22(3):488–97. doi: 10.5811/westjem.2021.3.49255 (PMC8203015; doi:10.5811/westjem.2021.3.49255)
Supplement: Supplementary file 1 [file wjem-22-488-s001.docx]

**Supplement 1. ICD*-9/10 codes reviewed.**

| ICD-9/10 code | Description |
| --- | --- |
| W3400XA | Accidental discharge from unspecified firearms or gun, initial encounter |
| W3400XS | Accidental discharge from unspecified firearms or gun, sequela |
| W3400XD | Accidental discharge from unspecified firearms or gun, subsequent encounter |
| W3301XA | Accidental discharge of shotgun, initial encounter |
| W320XXA | Accidental handgun discharge, initial encounter |
| W320XXD | Accidental handgun discharge, subsequent encounter |
| X93XXXA | Assault by handgun discharge, initial encounter |
| X93XXXD | Assault by handgun discharge, subsequent encounter |
| E9650 | Assault by handgun |
| X72XXXA | Intentional self-harm by handgun discharge, initial encounter |
| W3301XA | Accidental discharge of shotgun, initial encounter |
| Y35021A | Legal intervention involving injury by handgun, law enforcement official injured, initial encounter |
| E9220 | Accident caused by handgun |
| W320XXS | Accidental handgun discharge, sequela |
| X959XXA | Assault by unspecified firearm discharge, initial encounter |
| X749XXA | Intentional self-harm by unspecified firearm discharge, initial encounter |
| X749XXD | Intentional self-harm by unspecified firearm discharge, subsequent encounter |
| X959XXD | Assault by unspecified firearm discharge, subsequent encounter |
| X958XXA | Assault by other firearm discharge, initial encounter |
| Y249XXA | Unspecified firearm discharge, undetermined intent, initial encounter |
| X9501XA | Assault by airgun discharge, initial encounter |
| W3409XA | Accidental discharge from other specified firearms, initial encounter |
| Y240XXA | Airgun discharge, undetermined intent, initial encounter |
| W34010A | Accidental discharge of airgun, initial encounter |
| W34010D | Accidental discharge of airgun, subsequent encounter |
| W3312XA | Accidental malfunction of hunting rifle, initial encounter |
| X958XXS | Assault by other firearm discharge, sequela |
| X940XXD | Assault by shotgun, subsequent encounter |
| X731XXA | Intentional self-harm by hunting rifle discharge, initial encounter |
| W3302XA | Accidental discharge of hunting rifle, initial encounter |
| X940XXA | Assault by shotgun, initial encounter |
| X959XXS | Assault by unspecified firearm discharge, sequela |
| W3409XD | Accidental discharge from other specified firearms, subsequent encounter |
| W3319XA | Accidental malfunction of other larger firearm, initial encounter |

***ICD: International Classification of Diseases**
