# Supplementary material for: Gun Violence and Firearm Injuries in West Michigan: Targeting Prevention
Source: West J Emerg Med. 2021 May 19;22(3):488–97. doi: 10.5811/westjem.2021.3.49255 (PMC8203015; doi:10.5811/westjem.2021.3.49255)
Supplement: Supplementary file 2 [file wjem-22-488-s002.docx]

Supplement 2. Mechanism of injury comparisons.

| Characteristic | Attempted Murder/Bodily Harm n=213 | Accidental Discharge  n=64 | Self-inflicted  n=14 | p-value |
| --- | --- | --- | --- | --- |
| Age^@^ | 25 [19-31.5]^#^ | 22 [17.3-34.8]^^^ | 39 [25.7-62]^#,^^ | 0.011^#^; 0.008^^^ |
| Race, No. (%) |  |  |  | <0.001 |
| African American | 182 (85.4) | 27 (12.7) | 4 (1.9) |  |
| Caucasian | 31 (40.3) | 36 (46.8) | 10 (13) |  |
| Injury severity score^@^ | 1 [1- 9.3]^#, ^^ | 9 [2.5-14]^#^ | 21 [6.5-25] ^^^ | 0.03^#^; 0.002^^^ |
| LOS, survivors | 1 [1-3]^#^ | 1 [1-1]^#, ^^ | 2 [1-3] ^^^ | <0.001^#^; 0.007^^^ |
| Mortality, No. (%) | 15 (7) | 2 (3.1) | 7 (50) | <0.001 |
| Hospital charges^@^ | $10,184  [$3,314-$31,250] | $1,381  [$825-$10,041] | $19,508  [$10,849-$25,921] | <0.001 |
| Time of year, No. (%) |  |  |  | 0.54 |
| Fall | 43 (20.4) | 11 (17.2) | 2 (14.3) |  |
| Spring | 61 (28.9) | 17 (26.6) | 2 (14.3) |  |
| Summer | 62 (29.4) | 24 (37.5) | 4 (28.6) |  |
| Winter | 45 (21.3) | 12 (18.8) | 6 (42.9) |  |

^@^Median [interquartile range]

Superscripts #,^ denote the comparison between columns and their associated significant p-value.
